# BMI increase through puberty and adolescence is associated with risk of adult stroke

**DOI:** 10.1212/WNL.0000000000004158

**Published:** 2017-07-25

**Authors:** Claes Ohlsson, Maria Bygdell, Arvid Sondén, Christina Jern, Annika Rosengren, Jenny M. Kindblom

**Affiliations:** From the Centre for Bone and Arthritis Research, Institute of Medicine (C.O., M.B., J.M.K.), Bioinformatics Core Facility (A.S.), Institute of Biomedicine (C.J.), and Department of Molecular and Clinical Medicine (A.R.), the Sahlgrenska Academy at University of Gothenburg, Sweden.

## Abstract

**Objective:**

To evaluate the contribution of prepubertal childhood body mass index (BMI) and BMI change through puberty and adolescence, 2 distinct developmental BMI parameters, for risk of adult stroke in men.

**Methods:**

In this population-based study in Gothenburg, Sweden, men born in 1945–1961 with information on both childhood BMI at age 8 and BMI change through puberty and adolescence (BMI at age 20–BMI at age 8) were followed until December 2013 (n = 37,669). Information on stroke events was retrieved from high-quality national registers (918 first stroke events, 672 ischemic stroke events [IS], 207 intracerebral hemorrhage events [ICH]).

**Results:**

BMI increase through puberty and adolescence (hazard ratio [HR] 1.21 per SD increase; 95% confidence interval [CI] 1.14–1.28), but not childhood BMI, was independently associated with risk of adult stroke. Subanalyses revealed that BMI increase through puberty and adolescence was associated with both IS (HR per SD increase 1.19; 95% CI 1.11–1.28) and ICH (HR per SD increase 1.29; 95% CI 1.15–1.46). High BMI increase during puberty was strongly associated with increased risk of adult hypertension (odds ratio per SD increase 1.35; 95% CI 1.32–1.39).

**Conclusions:**

BMI increase through puberty and adolescence is associated with risk of adult IS and ICH in men. We propose that greater BMI increases during puberty contribute to increased risk of adult stroke at least partly via increased blood pressure.

Stroke incidence has decreased among older individuals yet it has been increasing among young adults.^1,2^ The cause for the increased stroke incidence in young adults is not known, but it coincides with the obesity epidemic.^1,3^

A high adult body mass index (BMI) is a risk factor for stroke^4^ and there is strong evidence of an association between a high BMI in young adulthood and risk of adult stroke in men.^5–8^ For prepubertal childhood BMI, there is no clear evidence of an association with adult risk of stroke.^9–11^ All these previous studies had only one BMI measurement available and could not separate the effect of BMI at childhood and the effect of BMI increase through puberty and adolescence for stroke risk.^12^

To evaluate the relative contribution of these 2 distinct developmental BMI parameters for the risk of adult stroke, at least one BMI measurement from each period is required. In the population-based BMI Epidemiology Study (BEST) in Gothenburg, Sweden, information on childhood BMI and young adult BMI shortly after puberty has been collected, and linkage to high-quality Swedish national registers has provided information on outcomes. Using this information, we recently demonstrated that excessive BMI increase during puberty is a risk marker of adult cardiovascular disease (CVD) mortality.^13^

We hypothesize that BMI increase during puberty might be a risk marker of adult stroke. The aim with the present study was therefore to evaluate the relative contribution of childhood BMI and BMI increase through puberty and adolescence as risk markers for adult stroke.

## METHODS

### Study population.

We collected birthweight as well as directly measured height and weight from centrally archived School Health Care (SHC) records for all men born 1945 to 1961 in Gothenburg, Sweden (see also e-Methods at Neurology.org). We also collected height and weight at young adult age from military conscription tests. Conscription was mandatory until 2008 for all Swedish men. The study cohort was linked to high-quality national disease registers using personal identity numbers (PINs) from the included participants. Eligible individuals were those with a SHC record in the central archive and a 10-digit PIN (figure e-1). Participants with data available for calculation of both childhood BMI and young adult BMI were included in the present study (n = 37,669; figure e-1 and table 1).

**Table 1:**
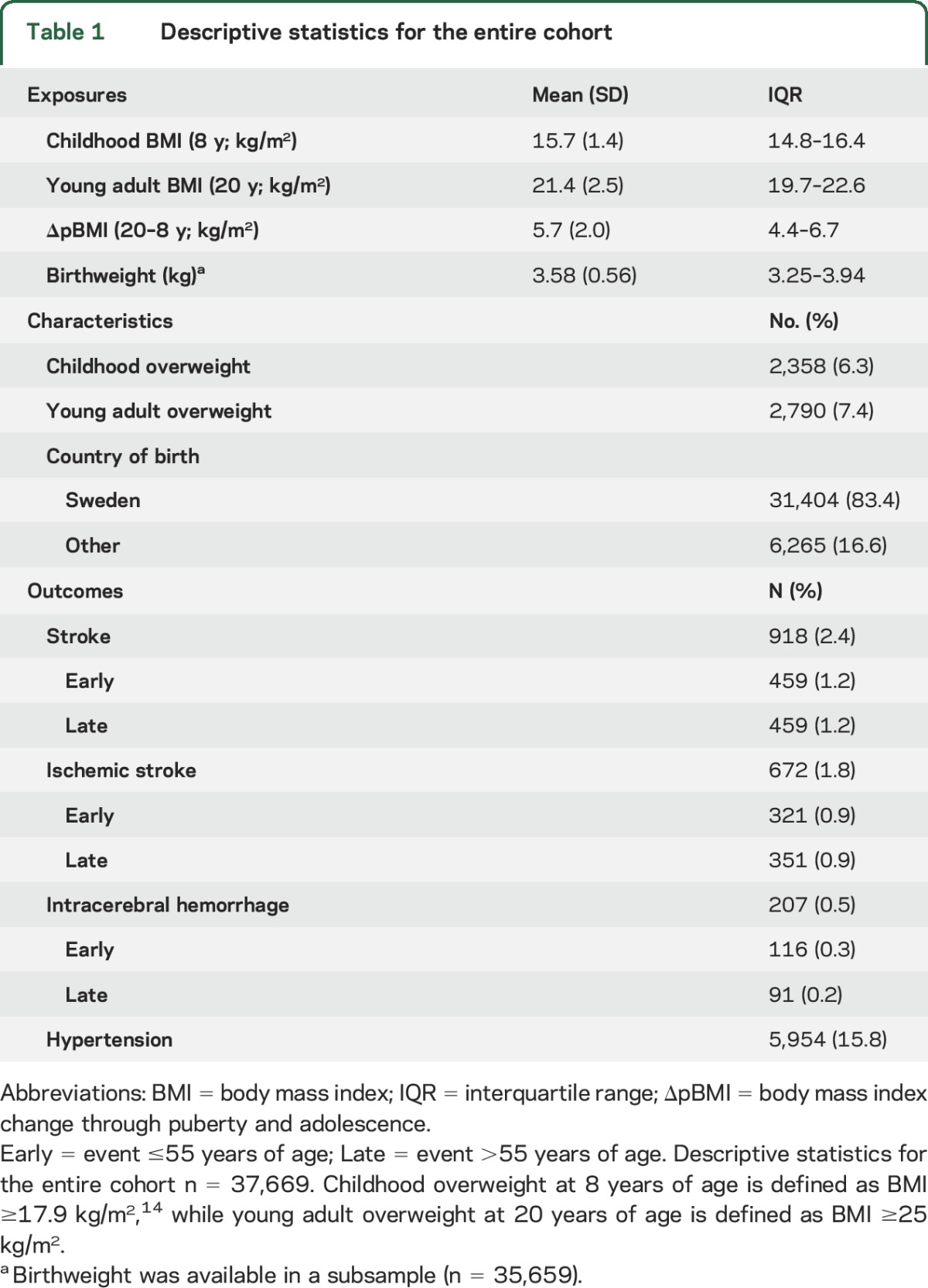
Descriptive statistics for the entire cohort

### Standard protocol approvals, registrations, and patient consents.

This study was approved by the ethics committee of the University of Gothenburg, Sweden. There was no commercial sponsorship.

### Exposures.

Prepubertal childhood BMI at 8 years of age and young adult BMI at 20 years of age were calculated using all paired height and weight measurements in the period between 6.5 and 9.5 years of age for prepubertal childhood BMI, and in the period 17.5–22 years of age for young adult BMI, and age-adjusted using linear regression models to 8 and 20 years of age, respectively.^13^ BMI change through puberty and adolescence was defined as the difference between young adult BMI and childhood BMI. Childhood overweight (≥17.9 kg/m^2^) and obesity (≥20.0 kg/m^2^) was defined according to the Centers for Disease Control and Prevention cutoff at 8 years of age^14^ and young adult overweight and obesity were defined as ≥25 kg/m^2^ and ≥30 kg/m^2^, respectively. Birthweight was retrieved from SHC records.

### Outcomes.

Linkage to registers held by the National Board of Health and Welfare and Statistics Sweden was performed using the individuals' 10-digit PIN. Dates and diagnoses for the first stroke event and first hypertension diagnosis were retrieved from high-quality national Swedish registers: the National Inpatient Register (as main diagnosis) and Cause of Death Register (as underlying cause of death; table e-1).^15–17^ The National Inpatient Register, including information on inpatient care diagnoses, was initiated in 1964 with full coverage in the Gothenburg region from 1972. The Cause of Death Register includes information on causes of death since 1961. The stroke events were defined according to the International Classification of Diseases system codes (table e-1). Participants who had a stroke, died, or emigrated before age 20 were excluded from the analysis (figure e-1). The men in the study were followed from 20 years of age until censoring due to stroke, death, migration, or until December 31, 2013, and were 52–68 years of age on December 31, 2013.

### Statistical analysis.

We used Cox proportional hazard regression to analyze the association between exposures and events. Childhood BMI was log-transformed and standardized and BMI change through puberty and adolescence was standardized when used in the Cox regression models. The assumption of proportionality was confirmed for all variables. Possible interactions were evaluated by addition of an interaction term in the linear Cox regression models, and *p* < 0.05 for the interaction term was interpreted as a statistically significant interaction. Possible nonlinear associations were evaluated by inclusion of a quadratic term and by using a restricted cubic spline approach.^18^ We used logistic regression to analyze the association between exposures and an adult diagnosis of hypertension. Kaplan-Meier survival plots, and analyses using restricted cubic splines, were done in R^19^ using the survival^20^ and rms^21^ packages. For all other statistical analyses, SPSS version 22 (SPSS Inc., Chicago, IL) was used.

## RESULTS

### Study cohort.

In this population-based study, men born in 1945–1961 with information on both childhood BMI at age 8 and BMI change through puberty and adolescence (BMI at age 20–BMI at age 8) were followed until December 2013 (n = 37,669; figure e-1, table 1). Mean follow-up starting from 20 years of age was 37.6 years (1,415,973 person-years of follow-up). A total of 918 first stroke events (672 ischemic stroke [IS] events, 207 intracerebral hemorrhage [ICH] events) occurred before the end of follow-up (table 1). Undetermined stroke events (n = 39) were included in stroke events but not in IS or ICH.

### BMI increase through puberty and adolescence is independently associated with stroke events.

In the Cox proportional hazards models adjusted for birth year and country of birth, BMI increase through puberty and adolescence (hazard ratio [HR] 1.21 per SD increase; 95% confidence interval [CI] 1.14–1.28), but not childhood BMI, was directly associated with risk of stroke when evaluated separately (table 2). Similar results were seen when childhood BMI and BMI change through puberty and adolescence were included together in the same model (table 2). The role for childhood BMI and BMI change through puberty and adolescence were then evaluated for stroke events divided into IS and ICH. BMI increase through puberty and adolescence was moderately associated with IS (HR 1.19 per SD increase; 95% CI 1.11–1.28) and strongly associated with ICH (HR 1.28 per SD increase; 95% CI 1.14–1.45; table 2), while childhood BMI was not associated with IS or ICH (table 2).

**Table 2:**
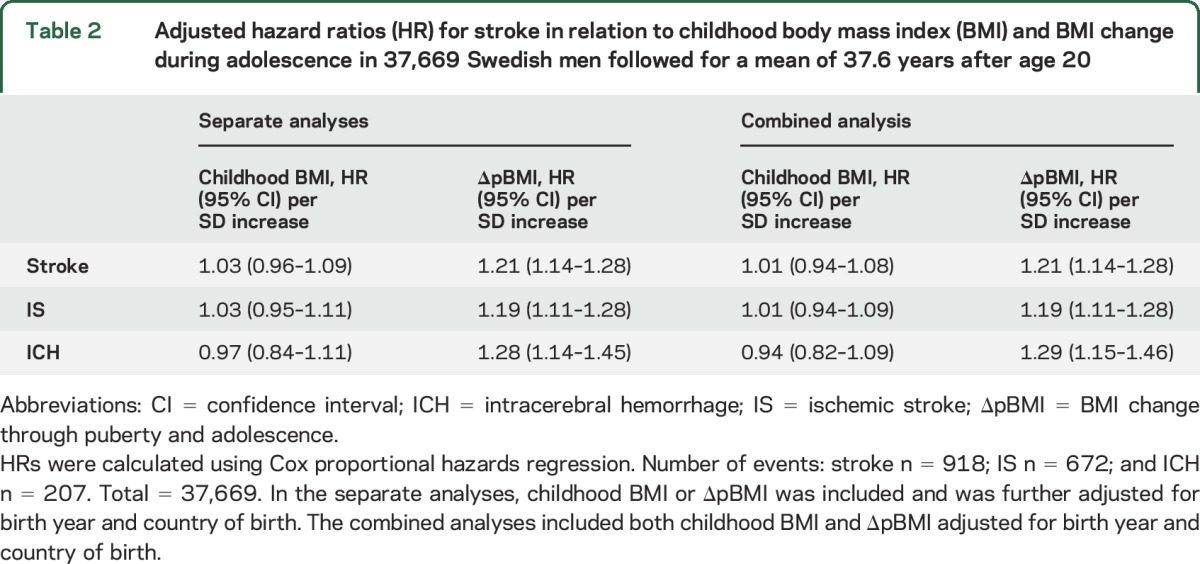
Adjusted hazard ratios (HR) for stroke in relation to childhood body mass index (BMI) and BMI change during adolescence in 37,669 Swedish men followed for a mean of 37.6 years after age 20

**Figure 1:**
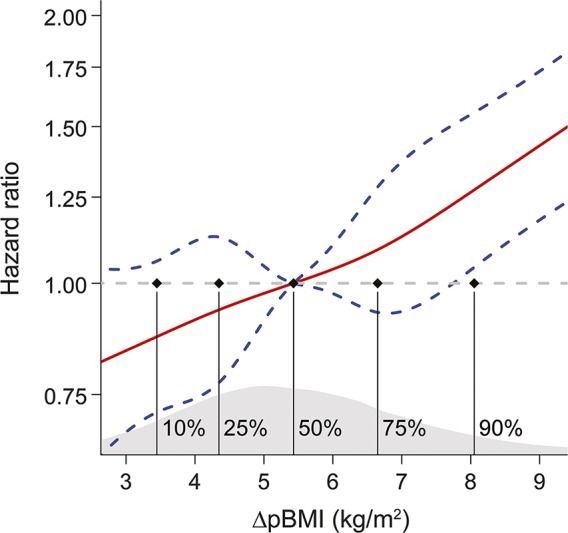
Hazard ratios (HRs) for stroke events according to body mass index (BMI) change through puberty and adolescence Cox regression analysis using a restricted cubic spline approach for a flexible nonlinear assessment of the HR for stroke events after 20 years of age in relation to BMI change through puberty and adolescence ΔpBMI (nonsignificant for nonlinearity). Five knots were placed at the ΔpBMI percentiles 10, 25, 50, 75, and 90 (indicated by vertical black lines). The model was adjusted for birth year and country of birth. Data presented as HR (red line) ± the 95% confidence interval (blue line). The distribution of participants according to ΔpBMI is shown in gray in the lower part of the figure. The horizontal dashed line corresponds to the reference (median ΔpBMI = 5.43 kg/m^2^) HR of 1.0 (no excess rate of events).

Both boys developing overweight during puberty (HR 1.81; 95% CI 1.41–2.33) and boys who were overweight consistently throughout childhood/puberty (HR 1.71; 95% CI 1.22–2.38) had increased risk of adult stroke compared with boys without childhood or young adult overweight (table 3). In contrast, participants who were overweight at 8 years of age and became normal weight during puberty did not have increased risk of adult stroke compared with participants with normal weight both at 8 years and at 20 years of age (table 3). Similar results were seen for IS and ICH, with the most pronounced effect for the association between overweight developed during puberty and ICH (HR 3.03; 95% CI 1.97–4.65; table 3). The same pattern was also seen in less powered analyses for obesity, with the strongest association seen for participants who developed obesity during puberty (table e-2). There was no evidence of a nonlinear association between BMI increase through puberty and adolescence and risk of adult stroke (figure 1), IS (figure e-2A), or ICH (figure e-2B). A Kaplan-Meier survival plot revealed increased risk of stroke for participants above the median BMI increase through puberty and adolescence compared to participants below the median (*p* < 0.001, figure e-3). There was no statistically significant interaction between childhood BMI and BMI increase through puberty and adolescence for the association with stroke events.

**Table 3:**
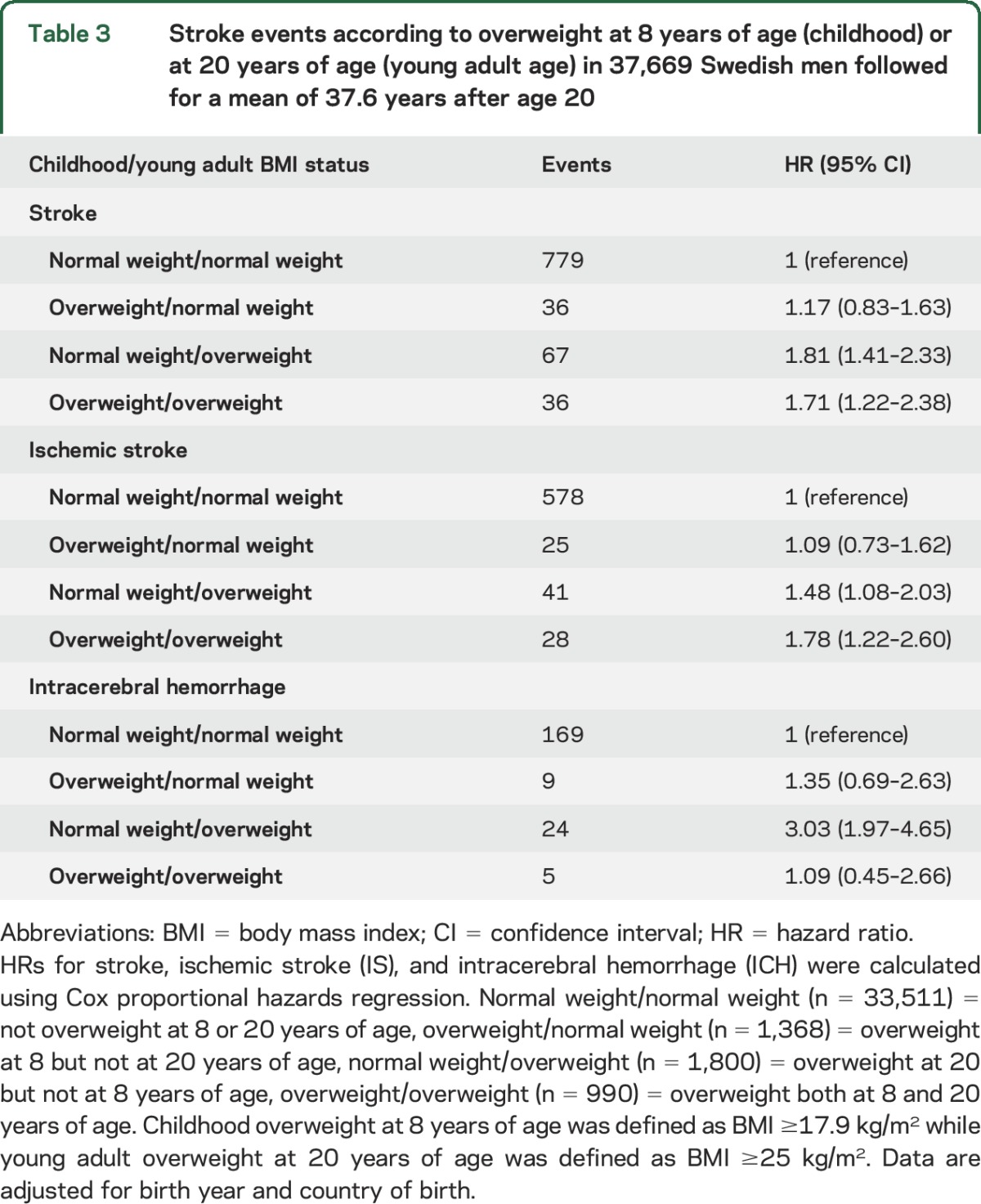
Stroke events according to overweight at 8 years of age (childhood) or at 20 years of age (young adult age) in 37,669 Swedish men followed for a mean of 37.6 years after age 20

As expected, young adult BMI was associated with risk of adult stroke (HR 1.18 per SD increase; 95% CI 1.11–1.26; table e-3). However, in combined analysis including both BMI change through puberty and adolescence and young adult BMI, only BMI increase through puberty and adolescence was associated with risk of adult stroke, demonstrating that BMI increase through puberty and adolescence is an independent risk marker of stroke (combined analysis, BMI increase through puberty and adolescence HR 1.20 per SD increase; 95% CI 1.07–1.35; young adult BMI HR 1.01 per SD increase; 95% CI 0.90–1.13; table e-3). Evaluation using cumulative incidence plots of stroke events and nonstroke mortality did not indicate that there was competing nonstroke mortality disturbing the present finding of increased risk of stroke for participants with a BMI change during puberty above the median compared with participants below the median of BMI change during puberty (figure e-4).

### Early and late stroke events.

Next we evaluated early (≤55 years of age) and late (>55 years of age) stroke events separately. These analyses revealed that BMI increase through puberty and adolescence adjusted for childhood BMI predicted early stroke (HR 1.22 per SD increase; 95% CI 1.12–1.32) and IS (HR 1.18; 95% CI 1.07–1.30; table 4). Importantly, the association between BMI increase through puberty and adolescence adjusted for childhood BMI and risk of early ICH was substantial (HR 1.35 per SD increase; 95% CI 1.16–1.56; table 4). BMI increase through puberty and adolescence was also associated with late stroke (HR 1.19 per SD increase; 95% CI 1.09–1.30) and IS (HR 1.19 per SD increase; 95% CI 1.08–1.32; table 4). Thus, greater BMI increase during puberty was a moderate risk marker of both early and late adult stroke and IS while it was a substantial risk marker specifically of early adult ICH.

**Table 4:**
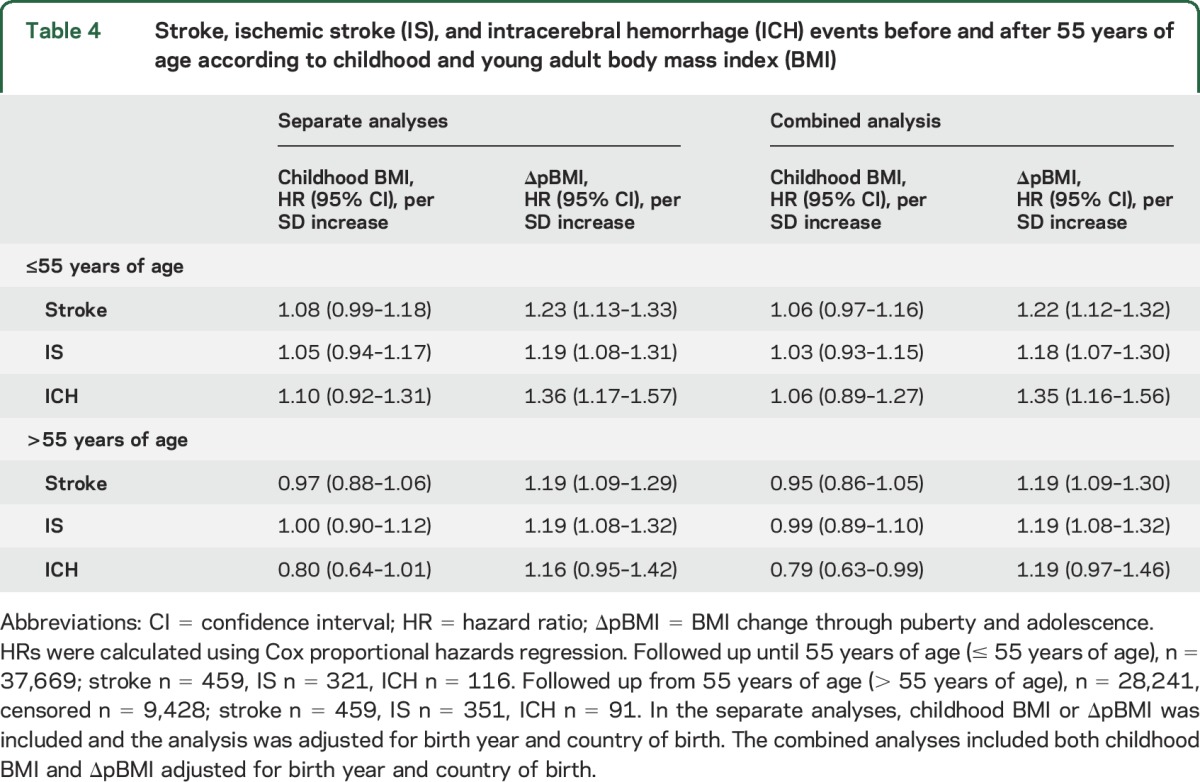
Stroke, ischemic stroke (IS), and intracerebral hemorrhage (ICH) events before and after 55 years of age according to childhood and young adult body mass index (BMI)

### Sensitivity analysis.

In order to control for disease-related weight loss, we performed sensitivity analyses for the associations with risk of stroke by exclusion of participants with censoring during the first 10 years of follow-up (n = 1,035). Exclusion of the first 10 years of follow-up resulted in similar results for the association between BMI increase through puberty and adolescence and risk of adult stroke (HR 1.21 per SD increase; 95% CI 1.14–1.29, adjusted for childhood BMI). We also evaluated a subpopulation including only boys born in Sweden and with parents born in Sweden. The described association for BMI increase through puberty and adolescence with young adult stroke events was similar in this subpopulation (n = 31,404; stroke HR 1.22 per SD increase; 95% CI 1.14–1.30).

### Adjustment for birthweight.

Birthweight was available in a subsample of the present cohort (n = 35,659). Additional adjustment for birthweight did not alter any of the associations. This could be illustrated by the fact that the association between BMI increase through puberty and adolescence and stroke was similar before (HR per SD increase 1.21; 95% CI 1.14–1.28) and after (HR per SD increase 1.20; 95% CI 1.13–1.28) adjustment for birthweight. No statistically significant interaction was seen between birthweight and BMI increase through puberty and adolescence for the association with risk of stroke.

### Association with hypertension.

As hypertension is a well-established risk factor for both IS and ICH, we next evaluated whether or not BMI increase through puberty and adolescence was associated with an adult diagnosis of hypertension using a logistic regression analysis. Interestingly, there was a strong association between BMI increase through puberty and adolescence and hypertension (OR 1.36 per SD increase; 95% CI 1.32–1.40). This association was similar when individuals with a stroke event (n = 918) were omitted from the analysis (OR 1.35 per SD increase; 95% CI 1.32–1.39).

## DISCUSSION

The reason for the recent unexpected trend of increased stroke incidence among young adult participants is unknown but it coincides with the obesity epidemic. We have recently demonstrated that BMI increase through puberty and adolescence is an independent predictor of adult CVD mortality.^13^ However, the relative role of childhood BMI and BMI increase through puberty and adolescence as risk markers for adult stroke is unknown.^12^ We herein demonstrate that BMI increase through puberty and adolescence, but not childhood BMI, is associated with risk of adult stroke and that this is observed both for IS and ICH.

Using the well-powered BEST Gothenburg Study with information on BMI both at childhood and in young adult age, we recently made the observation that the correlations between childhood BMI and BMI increase through puberty and adolescence were only marginal.^13^ These 2 distinct developmental BMI parameters therefore have the potential to contribute nonoverlapping information as risk markers for adult diseases. This notion is supported by our present finding that BMI increase through puberty and adolescence, but not childhood BMI, is associated directly with risk of adult stroke. The role of BMI increase through puberty and adolescence as a risk marker of adult stroke was illustrated by the fact that boys developing overweight during puberty, but not boys with childhood overweight that normalized during puberty, had increased risk of adult stroke compared with boys without childhood or young adult overweight.

The present finding that young adult BMI predicts risk of adult stroke confirms the results from several previous studies.^5–8,13,22^ Importantly, our combined models including both BMI increase through puberty and adolescence and young adult BMI revealed that only BMI increase through puberty and adolescence independently associated with risk of adult stroke, demonstrating that BMI increase through puberty and adolescence is an independent risk marker for adult stroke.

In a recent study, BMI was analyzed both in children at 3–19 years of age and later in the same participants at 30–40 years of age and the relative contribution of these 2 BMI parameters for adult CVD risk factors was evaluated. In that study, obese adults had increased risks of hypertension and carotid artery atherosclerosis regardless of BMI status during childhood and the risk of these outcomes among overweight or obese children who became nonobese by adulthood were similar to those among persons who were never obese. These findings indicate that childhood BMI status does not contribute beyond adult BMI status to adult risk of CVD,^23^ supporting the present study that BMI increase through puberty and adolescence but not childhood BMI is associated with risk of adult stroke. However, in the study by Juonala et al.,^23^ it was not possible to determine the association specifically for BMI change through puberty and adolescence as the childhood BMI measurement for a majority of the participants was not performed before onset of puberty but rather during puberty and the adult BMI measurement was performed up to about 25 years after puberty. In addition, the study did not have enough statistical power to evaluate stroke events.

Subanalyses in the present study revealed that the direct association between BMI increase through puberty and adolescence and risk of adult stroke was the result of an association with both risk of IS and ICH. A large BMI increase during puberty was a moderate risk marker of both early (<55 years of age) and late (≥55 years of age) adult IS while it was a substantial risk marker specifically of early adult ICH. Although IS and ICH to some extent have separate risk factors, high blood pressure is a major risk factor for both diseases^24^ and interventions that target hypertension are believed to reduce 75% of the excess risk of stroke associated with a high BMI in adults.^25^ Interestingly, greater BMI increase during puberty was, in the present study, a strong predictor of an adult diagnosis of hypertension, suggesting that high blood pressure might be a mediating factor for the association between BMI increase through puberty and adolescence and risk of adult stroke.

We speculate that a contributing reason for the recent unexpected increased stroke incidence among young adult participants might be caused by a secular trend of more pronounced BMI increase during puberty. The observational nature of our study precludes making conclusive statements about the observed associations but our findings can be useful for hypothesis generation. Based on our findings in the present study, we hypothesize that avoiding excessive BMI increase during puberty might reduce the risk of adult stroke and that one should consider monitoring adult blood pressure in men with excessive BMI increase during puberty.

The strengths of the present study include the large size of the cohort, the extended adult follow-up, the possibility to adjust for birthweight, and the population-based nature of the cohort. In addition, the Swedish national disease registers are of recognized high quality, which permits a near-complete follow-up of participants in the study and their diagnoses as reported by the health care provider.^15,26,27^ The study is well-powered and it is therefore possible to identify relatively small effects. An important limitation of the present study is that no information on childhood socioeconomic factors or education is available for the included men born as early as in 1945–1961. Furthermore, we could not control for several important risk factors (e.g., smoking, exercise, and serum levels of blood lipids) or for BMI at middle age. The present cohort includes primarily Caucasian men and, therefore, the results may have limited generalizability to other ethnicities. The age interval of the present cohort was 52–68 years at the end of follow-up (December 31, 2013) and thus stroke risk later in life could not be studied. It should be emphasized that since the present cohort was born in 1945–1961, the prevalences of obesity are lower (1.3% at 8 years of age and 0.9% at 20 years in the present study), while the corresponding figures in more recent years are higher, 7.9% at 8 years of age^28^ and 3.8% at age 20 in a study from the military service conscription register.^29^ Thus the present study predates the childhood obesity epidemic and today's obesogenic environment may further enhance the observed associations with adult stroke events.

BMI increase through puberty and adolescence is associated with risk of adult IS and early adult ICH in men. We propose that high BMI increase during puberty might cause increased risk of adult stroke at least partly via increased blood pressure.

## Supplementary Material

Data Supplement

Accompanying Editorial

## GLOSSARY

BEST: BMI Epidemiology Study
BMI: body mass index
CI: confidence interval
CVD: cardiovascular disease
HR: hazard ratio
ICH: intracerebral hemorrhage
IS: ischemic stroke
PIN: personal identity number
SHC: School Health Care
