# Supplementary material for: BMI increase through puberty and adolescence is associated with risk of adult stroke
Source: Neurology. 2017 Jul 25;89(4):363–9. doi: 10.1212/WNL.0000000000004158 (PMC5574671; doi:10.1212/WNL.0000000000004158)
Supplement: Data Supplement [file supp_WNL.0000000000004158_Supplementary_Material.pdf]

Supplementary Methods

Study Population

For almost 100 years, the general health and wellbeing of Swedish children has been followed by school health care (SHC) from school start (at age 7 for the study cohort) throughout the school years. The SHC program includes vaccinations and direct measurements of height and weight performed by specially trained school nurses. From the early 1950s (coinciding with childhood measurements from ≈ birth year 1943), 98.5% of all pupils nationwide were covered by SHC (1). The participants had on average 1.9 measurements in the childhood BMI period and 1.3 measurements in the young adult BMI period. Information on country of birth and migration was retrieved from the Longitudinal Integration Database for Health Insurance and Labor Market Studies held at Statistics Sweden. Country of birth was categorized as Sweden (the study subject and both his parents born in Sweden) or not Sweden (the subject, or one or both parents not born in Sweden or information on country of birth is missing).

Exposures

Age adjustments of pre-pubertal childhood BMI (at age 8) and young adult BMI (at age 20) were performed separately using linear regression with BMI as dependent variable and age as independent variable using all available BMI measurements in the age intervals. The age intervals were 6.5- 9.5 years of age for pre-pubertal childhood BMI and 17.5-22 years of age for young adult BMI, respectively. The age-dependent change was assumed to follow the slope of the fitted models and BMI at eight / twenty years of age was estimated using the slope of the fitted model. Thus, the BMI values were interpolated on the population level. Onset and duration of puberty varies between individuals, and pubertal development greatly alters body composition (2). To avoid the confounding effect of ongoing puberty on BMI, we defined the pubertal period with a rather wide time window. Therefore, the time between 8 and 20 years included not only the complete pubertal period but also periods of varying length both before and after puberty. The BMI increase through puberty and adolescence was therefore defined as the difference between BMI at 20 years of age and BMI at 8 years of age.

Cohort representativeness

Sweden has had a compulsory school attendance since 1936, starting from the year the child turned 7. From the early 1950s, 98.5% of all children were followed by school health care (1). Due to missing data and missing Personal Identity Number, not all children with a school health record are included in the present study (24.7 % not included; Figure e-1). In order to investigate if those included in the study and those not included differ with regard to young adult BMI, we evaluated BMI from the examination at conscription. Young adult BMI at conscription was similar for the two groups (BMI mean [SD]; Included participants 21.13 [2.53] kg/m^2; Not included participants 21.15 [2.57]; non-significant using t-test), suggesting that the cohort is representative for Gothenburg, the second largest city in Sweden.

Statistical Analyses

There were no missing values for the main parameters (childhood BMI, BMI increase through puberty and adolescence, young adult BMI, country of birth, birth year or follow-up). The only parameter without a complete set of data was birthweight (n=35,659 which corresponds to 95% of the entire cohort). Models including birthweight only included the subgroup of boys with birthweight available. The assumption of proportionality in the Cox regression models was assessed both through visual evaluations of Schoenfeld residual plots, and through proportional hazard tests using the "survival" package in the R statistical software (5-7).

Childhood BMI was standardized within the study population, having zero mean and unit variance. The standard score (Z) was calculated as $Z=(x-\mu)/\sigma$. μ is the mean of the study population and σ is the standard deviation of the study population.

Cumulative incidence plots (Figure e-4) were performed for both stroke events and non-stroke mortality according to if participants had a BMI increase through puberty and adolescence above or below the median in BMI increase through puberty and adolescence.

References

1. Herlitz CW. Skolhälsovårdens historia. Bergvalls, Stockholm; 1961.
2. Loomba-Albrecht LA, Styne DM. Effect of puberty on body composition. Curr Opin Endocrinol Diabetes Obes. 2009;16(1):10-5.
3. Michaelsson K, Wolk A, Langenskiold S, Basu S, Warensjo Lemming E, Melhus H, et al. Milk intake and risk of mortality and fractures in women and men: cohort studies. BMJ (Clinical research ed). 2014;349:g6015.
4. Tunstall-Pedoe H, Kuulasmaa K, Amouyel P, Arveiler D, Rajakangas AM, Pajak A. Myocardial infarction and coronary deaths in the World Health Organization MONICA Project. Registration procedures, event rates, and case-fatality rates in 38 populations from 21 countries in four continents. Circulation. 1994;90(1):583-612.
5. R Core Team: R: A language and environment for statistical computing. R Foundation for Statistical Computing, Vienna, Austria, 2015. URL https://www.R-project.org/.
6. Therneau T: A Package for Survival Analysis in S. version 2.38, 2015. http://CRAN.R-project.org/package=survival.
7. Frank E Harrell Jr: rms: Regression Modeling Strategies. R package version 4.4-2, 2016. https://CRAN.R-project.org/package=rms.

Supplementary Tables

Table e-1 Definition of events according to the ICD system.

Outcome	*ICD10*	*ICD9*	*ICD8*
All stroke	I61, I63, I64	431, 433, 434, 436	431, 433, 434, 436
Ischemic stroke (IS)	I63	433, 434	433, 434
Intracerebral Hemorrhage (ICH)	I61	431	431
Hypertension	I10-15	401-405	400-404

Definition of outcomes in the present study according to the International Classification of Diseases 8 (ICD8, used during the years 1969-1986), ICD9 (1987-1996) and ICD10 (1997 and onwards). Information on events (diagnoses and deaths) was retrieved form The National Inpatient register (main diagnosis) and Cause of Death register (underlying cause of death). Information on hypertension was retrieved form The National Inpatient register (main diagnosis or additional diagnosis, in- or outpatient).

Table e-2 Stroke events according to obesity at 8 years of age (childhood) and/or at 20 years of age (young adult age) in 37,669 Swedish men followed for a mean of 37.6 years after age 20.

Childhood/Young adult BMI status	Events	HR (95% CI)
**Stroke**		
Normal weight/Normal weight	887	1 (reference)
Obesity/Normal weight	15	1.67(1.00-2.78)
Normal weight/Obesity	12	2.73(1.54-4.83)
Obesity/Obesity	4	1.80(0.68-4.82)

Hazard Ratios (HRs) for stroke were calculated using Cox proportional hazards regression. Normal weight/Normal weight (n=36,946)= Not obesity at 8 or 20 years of age, Obesity/Normal weight (n=395) = Obesity at 8 but not at 20 years of age, Normal weight/Obesity (n=219) = Obesity at 20 but not at 8 years of age, Obesity/Obesity (n=109) = Obesity both at 8 and 20 years of age. Childhood obesity at 8 years of age (n=504, 1.3%) was defined as BMI ≥20.03 kg/m^2 while young adult obesity at 20 years of age (n=328, 0.9%) was defined as BMI ≥30 kg/m^2. Data are adjusted for birth year and country of birth.

Table e-3 Adjusted Hazard Ratios for stroke in relation to BMI increase through puberty and adolescence and young adult BMI in 37,669 Swedish men followed for a mean of 37.6 years after age 20.

Outcome	*Separate analysis*		*Combined analysis*	
	ΔpBMI<br>HR (95% CI)<br>per SD increase	Young adult BMI<br>HR (95% CI)<br>per SD increase	ΔpBMI<br>HR (95% CI)<br>per SD increase	Young adult BMI<br>HR (95% CI)<br>per SD increase
Stroke	1.21(1.14-1.28)	1.18(1.11-1.26)	1.20(1.07-1.35)	1.01(0.90-1.13)
IS	1.19(1.11-1.28)	1.17(1.09-1.26)	1.17(1.03-1.34)	1.02(0.89-1.17)
ICH	1.28(1.14-1.45)	1.20(1.05-1.37)	1.45(1.13-1.85)	0.87(0.67-1.12)

Hazard Ratios (HRs) were calculated using Cox proportional hazards regression. IS= Ischemic stroke, ICH= Intracerebral Hemorrhage. ΔpBMI = BMI increase through puberty and adolescence, CI= Confidence Interval, SD= Standard Deviation. N=37,669. The analyses included both BMI increase through puberty and adolescence and young adult BMI adjusted for birth year and country of birth.

Figure e-1 Flow chart of included individuals. N= 37,669 Swedish men followed for a mean of 37.6 years.

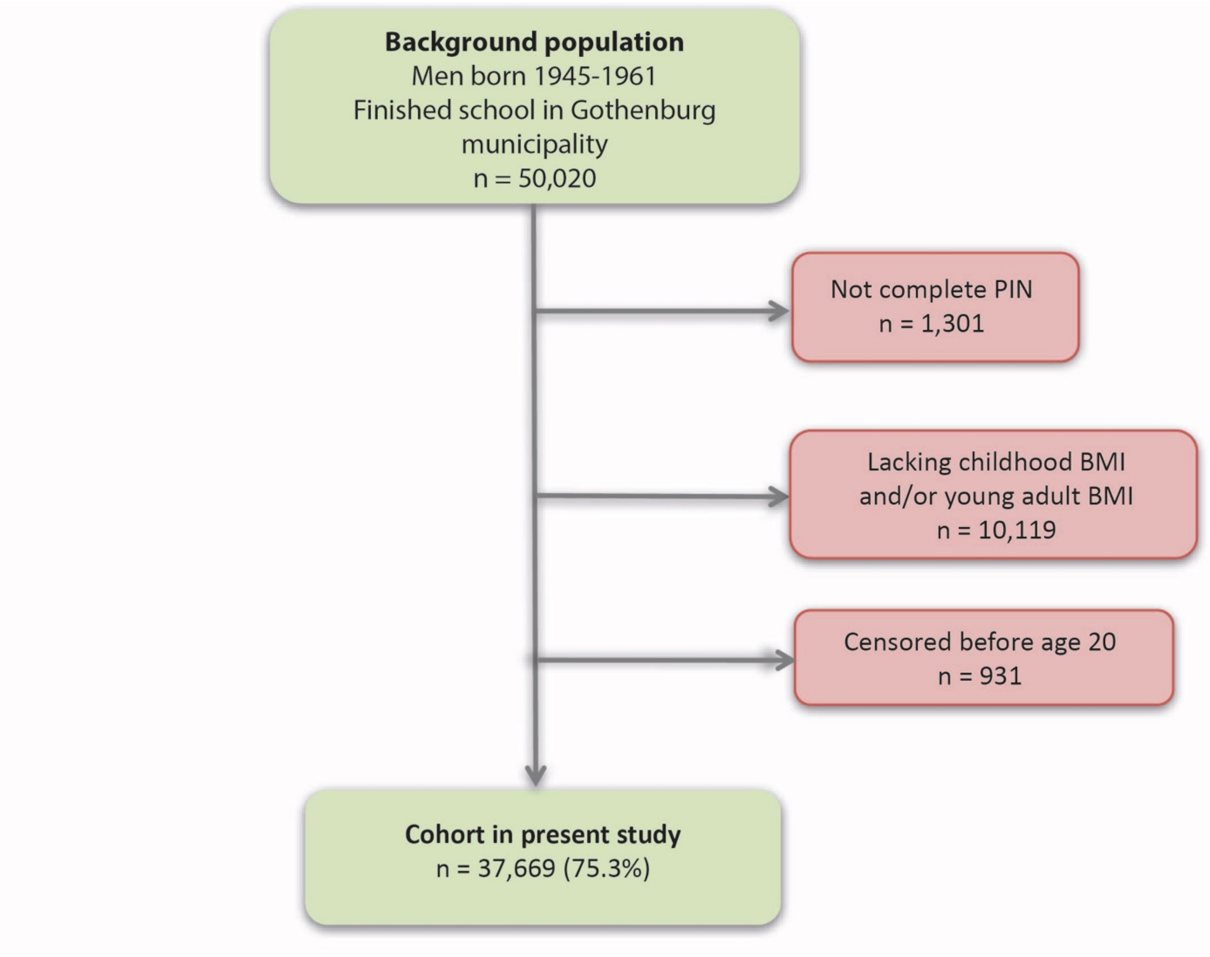

PIN= personal identity number, BMI= body mass index.

Figure e-2 Smoothed plots of hazard ratios (HRs) for ischemic stroke and intracerebral hemorrhage according to BMI increase through puberty and adolescence in 37,669 Swedish men followed for a mean of 37.6 years after age 20.

Figure e-2a

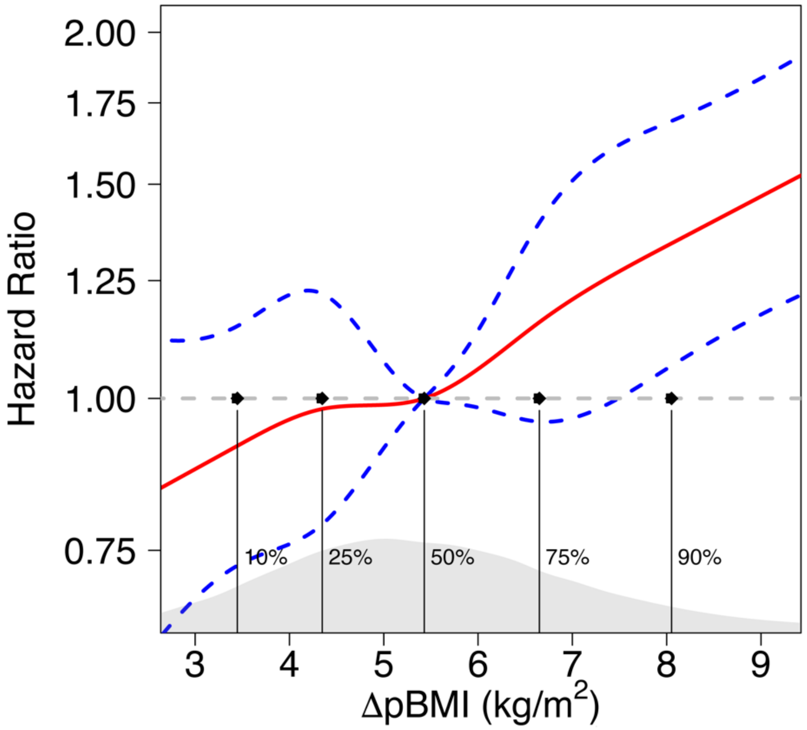

Figure e-2b

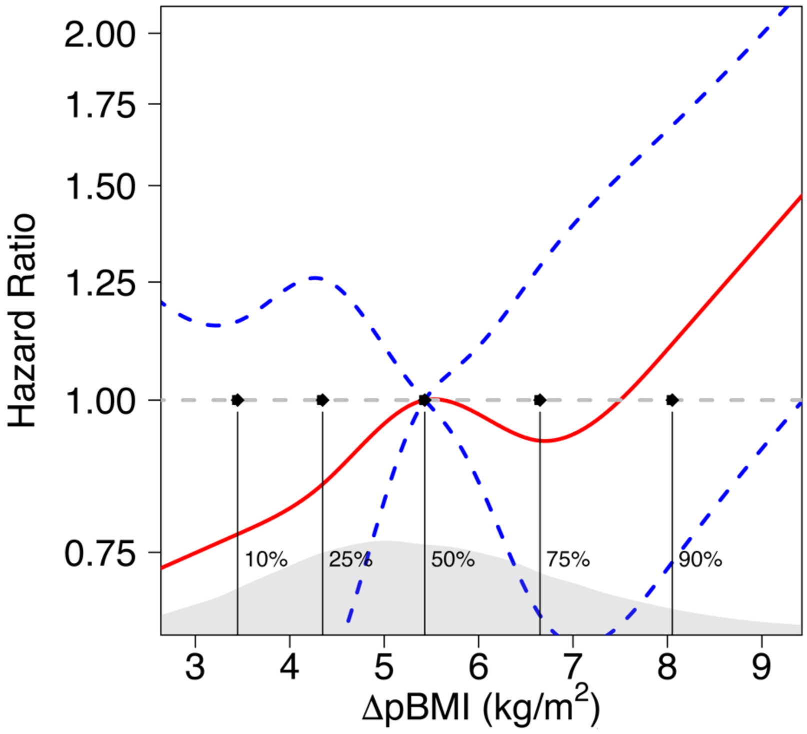

Cox regression analysis using a restricted cubic spline-approach for a flexible non-linear assessment of the hazard ratio (HR) for risk of ischemic stroke (A) and intracerebral hemorrhage (B) after 20 years of age in relation to BMI increase through puberty and adolescence (ΔpBMI; non-significant for non-linearity). Five knots were placed at the ΔpBMI percentiles 10, 25, 50, 75 and 90 (indicated by vertical black lines). The models were adjusted for birth year and country of birth. Data is presented as hazard ratio (red line) ± the 95% confidence interval (blue dotted line). The distribution of participants according to ΔpBMI is shown in gray in the lower part of the figure. The horizontal dashed line corresponds to the reference (median ΔpBMI= 5.43 kg/m^2) HR of 1·0 (no excess rate of events).

Figure e-3 Kaplan-Meier curve of stroke free survival according to BMI increase through puberty and adolescence in 37,669 Swedish men followed for a mean of 37.6 years after age 20.

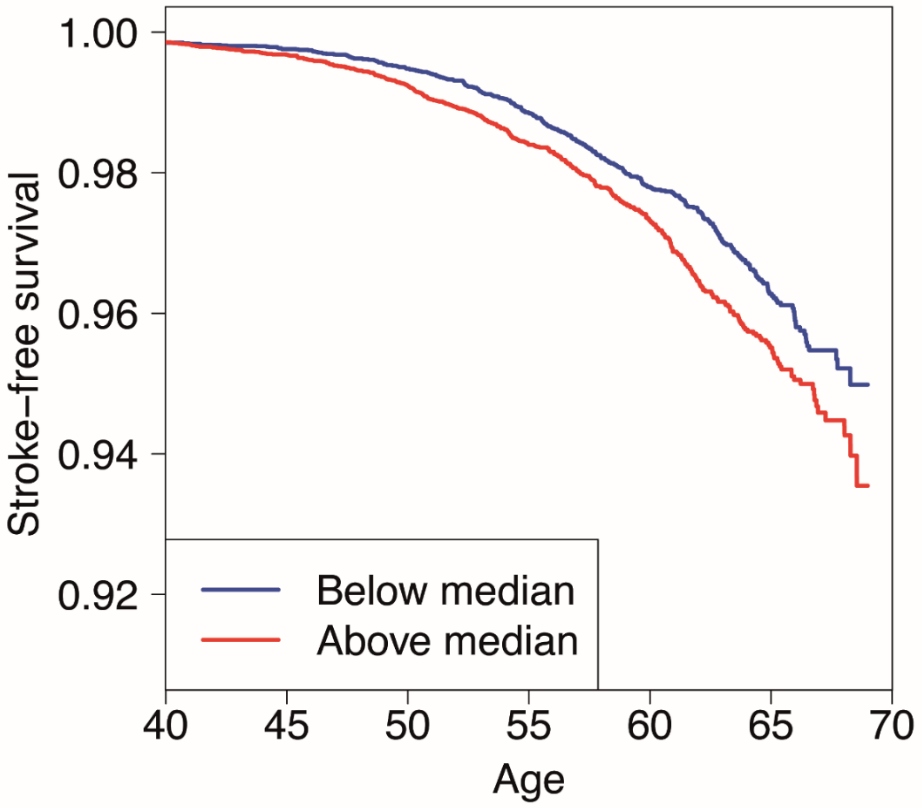

Below median						
**No at risk**	**17706**	**17358**	**16940**	**14568**	**9045**	**3142**
**Cum stroke events**	**27**	**43**	**91**	**192**	**319**	**406**
Above median						
**No at risk**	**17677**	**17337**	**16918**	**13670**	**7226**	**2615**
**Cum stroke events**	**26**	**57**	**132**	**267**	**377**	**470**

The graph shows stroke free survival according to if participants had a BMI increase through puberty and adolescence (ΔpBMI) above or below the median. The p value for comparison between the two groups assessed by log-rank test was $p < 0.001$.

Figure e-4 Cumulative incidence plots for stroke events (A) and non-stroke mortality (B).

Figure e-4a

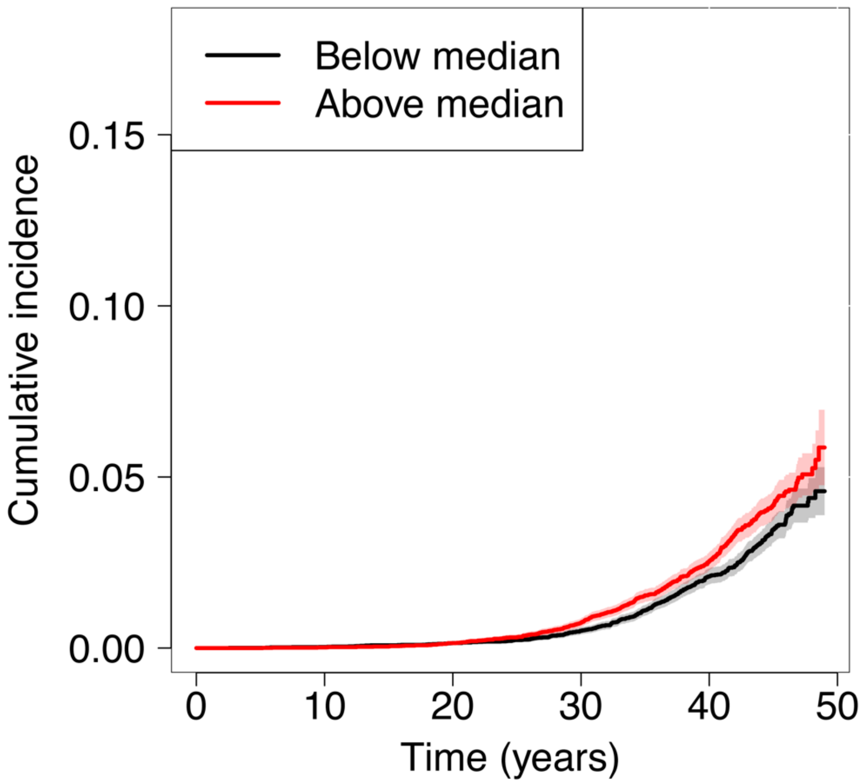

Figure e-4b

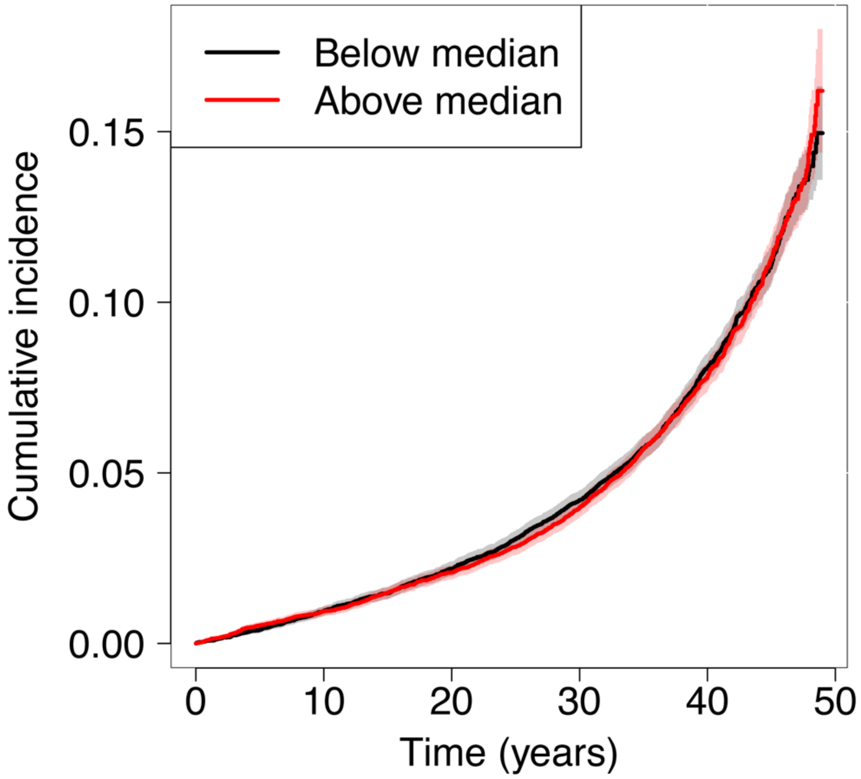

Stroke events (A) and non-stroke mortality (B) are shown according to if participants had a BMI increase through puberty and adolescence above (red) or below (black) the median. Data is shown as percent with events, and shaded areas represent 95% confidence intervals**.**
