# Supplementary material for: BMI increase through puberty and adolescence is associated with risk of adult stroke
Source: Neurology. 2017 Jul 25;89(4):363–9. doi: 10.1212/WNL.0000000000004158 (PMC5574671; doi:10.1212/WNL.0000000000004158)
Supplement: Accompanying Editorial [file supp_89_4_363_v2_index.html]

Accompanying Editorial 

# BMI increase through puberty and adolescence is associated with risk of adult stroke

## Accompanying Editorial

**Neurology® data supplements are not copyedited before publication. Published editorials and translations have been copyedited.  
 © 2017 American Academy of Neurology.  
  
 Files in this Data Supplement:**

- Accompanying Editorial - PDF
