# Supplementary material for: BMI increase through puberty and adolescence is associated with risk of adult stroke
Source: Neurology. 2017 Jul 25;89(4):363–9. doi: 10.1212/WNL.0000000000004158 (PMC5574671; doi:10.1212/WNL.0000000000004158)
Supplement: Accompanying Editorial [file supp_WNL.0000000000004158_312.pdf]

EDITORIAL

# Adolescent weight gain confers long-term increased stroke risk

## Weighty matters

Kathryn M. Rexrode, MD, MPH
Sue Y.S. Kimm, MD, MPH, SM

Correspondence to Dr. Rexrode: krexrode@partners.org

*Neurology*® 2017;89:312–313

Adult obesity has been consistently associated with risk of total, ischemic, and hemorrhagic stroke[1–3]; however, the degree to which change in weight and body mass index (BMI) during late childhood and adolescence affects adult risk of stroke has remained uncertain. Understanding this relationship is of particular importance given the rising obesity epidemic during childhood and adolescence.

Ohlsson et al.[4] in this issue of *Neurology*® provide key data on the long-term increased risk of weight gain during puberty, and the detrimental effects of overweight and obesity during adolescence and early adulthood on adult stroke risk. The authors analyzed BMI from childhood (age 8) through early adulthood (age 20) and the change over that period with risk of subsequent stroke in 36,000 Swedish men using high-quality school and military BMI records as well as health system linkage data. Each BMI change of 2 $kg/m^2$ from age 8 to age 20 was associated with a 21% increased risk of total stroke in adulthood. Risk of intracerebral hemorrhage was increased by 28% for each 2 $kg/m^2$ change, while risk of ischemic stroke increased by 19%. Notably, BMI gain (2 $kg/m^2$) during puberty/adolescence was associated with a 35% increased risk of adult hypertension, a likely mediating factor.

Boys who were overweight at both age 8 and age 20, as well as those who became overweight by age 20, had increased risk of stroke compared to those with normal weights. However, importantly, boys who were overweight at age 8 but who achieved normal weight at age 20 did not have increased risk of stroke during adulthood. Strengths of this thoughtful study include direct measures of height and weight from school and military records and long-term stroke follow-up. One limitation is the lack of data on women.

This study highlights the importance of events during adolescence on the later risk of chronic diseases and provides a potential explanation for the recently reported uptick in stroke incidence in midlife.[5] Despite being the most dynamic period biologically and behaviorally in our life cycle, adolescence is a relatively quiescent time in medical surveillance, in part because the adolescent has fewer common childhood infections and fewer immunization requirements. This age group often falls between pediatric and adult medicine.

Over the last 3 decades, obesity has reached a pandemic level globally, affecting all ages, especially in the United States. In recent years, the upward trajectory in young children (ages 2–5) seems to have stabilized.[6] However, obesity rates in older children (ages 6–11) and adolescents (ages 12–19) have continued upward. Between 1988–1989 and 2014–2015, adolescents doubled their obesity prevalence from 10.5% to 20.5% while older children had a 1.5-fold (11.3%–17.4%) increase. Furthermore, extreme obesity quadrupled among adolescents from 2.6% to 9.1%; for older children, the change was from 3.6%–4.3%. Based on the data from Ohlsson et al., this exponential increase in obesity in adolescents portends serious health consequences later in life.

Although the factors behind these trends remain incompletely understood, tantalizing clues exist. A large 10-year cohort study showed a breathtaking decline in physical activity during adolescence, with a drop of 100% and 64% in the physical activity median scores in black and white girls, respectively.[7] Further, a 10-year follow-up in the same cohort demonstrated a clear inverse relationship between physical activity and BMI changes.[8] Black women also became heavier than white women, although both groups doubled their rates of obesity, to 37% for black women and 18% for white women by age 19.[9]

The strong association between overweight status in early adulthood and BMI gain during adolescence and subsequent stroke risk offers one potential explanation for the recently reported dramatic risk in stroke rates among men and women in midlife (age 40–54).[5] Stroke prevalence among women aged 35–54 years tripled in the United States over the last 2 decades, corresponding to the rise in obesity rates.[5]

The role of extreme obesity, especially during adolescence, remains to be explored. The study by

See page 363

From the Division of Preventive Medicine (K.M.R.), Harvard Medical School, Brigham and Women's Hospital, Boston, MA; and Consultant in Epidemiology (S.Y.S.K.), Santa Fe, NM.

Go to Neurology.org for full disclosures. Funding information and disclosures deemed relevant by the authors, if any, are provided at the end of the editorial.


© 2017 American Academy of Neurology
© 2017 American Academy of Neurology. Unauthorized reproduction of this article is prohibited.

Ohlsson et al. did not stratify risk ratios by degrees of obesity due to low prevalence in this cohort. The transitioning into extreme obesity during adolescence is a concern, especially given its high incidence. The data from Ohlsson et al. suggest a dose-response relationship between the degree of obesity and stroke risk. However, there might be a threshold level of obesity beyond which the relationship may change from linear to exponential. These hypotheses require further investigation; we commend Ohlsson et al. for a study that leads to these interesting hypotheses.

The report by Ohlsson et al.[3] demonstrates the critical import of changes in BMI during adolescence, an oft-overlooked phase of the life cycle. Importantly for public health implications, children who reduced their BMI to the normal range by age 20 had no long-term increased risk of stroke. These findings emphasize the need to target interventions for children and adolescents to prevent overweight and obesity in early adulthood and also reduce future cardiovascular morbidity. Battling childhood and adolescent obesity is the first step toward prevention of stroke and major adult chronic diseases.


## STUDY FUNDING

No targeted funding reported.


## DISCLOSURE

The authors report no disclosures relevant to the manuscript. Go to Neurology.org for full disclosure forms.

## REFERENCES


1. Rexrode KM, Hennekens CH, Willett WC, et al. A prospective study of body mass index, weight change, and risk of stroke in women. JAMA 1997;277:1539–1545.
2. Kurth T, Gaziano JM, Berger K, et al. Body mass index and the risk of stroke in men. Arch Intern Med 2002;162: 2557–2562.
3. Strazzullo P, D'Elia L, Cairella G, Garbagnati F, Cappuccio FP, Scalfi L. Excess body weight and incidence of stroke: meta-analysis of prospective studies with 2 million participants. Stroke 2010;41:e418–e426.
4. Ohlsson C, Bygdell M, Sondén A, Jern C, Rosengren A, Kindblom JM. BMI increase through puberty and adolescence is associated with risk of adult stroke. Neurology 2017;89:363–369.
5. Towfighi A, Zheng L, Ovbiagele B. Weight of the obesity epidemic: rising stroke rates among middle-aged women in the United States. Stroke 2010;41:1371–1375.
6. Carroll MD, Lawman HG, Fryar CD, et al. Trends in obesity prevalence among children and adolescents in the United States, 1988–1994 through 2013–2014. JAMA 2016;315:2292–2299.
7. Kimm SYS, Glynn NW, Kriska AM, et al. Decline in physical activity in black girls and white girls during adolescence. N Engl J Med 2002;347:709–715.
8. Kimm SYS, Glynn NW, Obarzanek E, et al. The longitudinal relation between changes in physical activity and body mass index during adolescence. Lancet 2005;366: 301–307.
9. Kimm SYS, Barton BA, Obarzanek E, et al. Racial divergence in adiposity during adolescence: the NHLBI Growth and Health Study. Pediatrics 2001; 107:e34.




© 2017 American Academy of Neurology. Unauthorized reproduction of this article is prohibited.
